# A Cross-Sectional Study on Cognitive Vulnerability Patterns in Dental Anxiety: The Italian Validation of the Dental Fear Maintenance Questionnaire (DFMQ)

**DOI:** 10.3390/ijerph20032298

**Published:** 2023-01-28

**Authors:** Roberta Gasparro, Federica Di Spirito, Mario Cangiano, Arianna De Benedictis, Pasquale Sammartino, Gilberto Sammartino, Vincenzo Bochicchio, Nelson Mauro Maldonato, Cristiano Scandurra

**Affiliations:** 1 Department of Neuroscience, Reproductive Science and Dentistry, University of Naples Federico II, 80131 Naples, Italy; 2 Department of Medicine, Surgery and Dentistry, University of Salerno, 84084 Fisciano, Italy; 3 Multidisciplinary Department of Medical-Surgical and Odontostomatological Specialties, University of Campania “Luigi Vanvitelli”, 80138 Naples, Italy; 4 Department of Humanistic Studies, University of Calabria, 87036 Cosenza, Italy

**Keywords:** dental anxiety, cognition, perception, aged

## Abstract

Dental anxiety is a crucial problem for dentistry because it may represent a significant risk to oral health. Within the framework of the Cognitive Vulnerability Model (CVM), which sheds light on the cognitive vulnerability patterns that may cause dental anxiety, this study aimed to assess the psychometric characteristics of the Italian version of the Dental Fear Maintenance Questionnaire (DFMQ). The DFMQ is a 32-item questionnaire that assesses four cognitive vulnerability patterns, i.e., dangerousness, disgust, unpredictability, and uncontrollability. In a sample of 200 dental patients who had accessed public-university-hospital dental surgery, this study assessed the model fit of the DFMQ and different types of validity (i.e., predictive, convergent, construct, and discriminant validity). In addition, potential differences between DFMQ dimensions were assessed based on gender (men vs. women) and age ranges. All indicators of cognitive vulnerability were significantly associated with high dental anxiety when each variable was included independently. In contrast, when the DFMQ subscales were considered together, only unpredictability and uncontrollability were found to be associated with high dental anxiety. Women had higher scores for unpredictability, uncontrollability, and general dangerousness than men. In addition, older patients had higher scores for some vulnerability cognitive patterns than younger patients. This study provides Italian dentists and researchers with a valid questionnaire to assess cognitive factors associated with dental anxiety.

## 1. Introduction

Dental anxiety is a common psychological state among dental patients that can be defined as an emotional state of suffering or apprehension in anticipation of dental treatment [1]. Nevertheless, sometimes the level of anxiety is so excessive as to induce dental phobia, low compliance, or disabling levels of psychological distress that can be a serious risk to oral health or, in extreme cases, a significant barrier to access dental care [2,3,4,5].

According to Beaton and collaborators [6], the factors that cause dental anxiety may be exogenous (e.g., painful or traumatic dental experiences), endogenous (e.g., personality traits), or indirect (e.g., negative dental experiences told by significant others or heard through the media). The epidemiological research in western contexts has reported that a percentage ranging from 10% to 20% of adults suffer from high levels of dental anxiety [7,8].

While some studies have attributed to past traumatic dental episodes (i.e., non-cognitive factors) a central pathogenetic role in terms of the development of dental anxiety [9,10], other studies have highlighted the role of certain subjective factors in the processing of dental stimuli (i.e., cognitive factors), including the patient’s personal perceptions with regard to the dentist and dental treatment [1,11]. For example, Carrillo-Diaz and collaborators [12] reported that cognitive factors were the strongest predictors of dental anxiety and that, once cognitive factors were included in a regression model, non-cognitive factors lost their predictive power.

In this regard, the Cognitive Vulnerability Model (CVM) is the most used theoretical model concerning cognitive factors associated with dental anxiety [13]. According to the CVM, the central dimension associated with dental anxiety is represented by the “vulnerability patterns,” which are cognitive schemas that can be activated when the patient is exposed to dental stimuli, independently of past dental traumatic events. The contents of these schemas involve four interconnected perceptions of the dental event, designated as uncontrollable, unpredictable, potentially dangerous or harmful, and disgusting. Once a schema is activated, two processes take place simultaneously. These are an immediate and automatic affective response (i.e., fear) and a cognitive assessment of the event. Armfield [1] reported that perceptions related to vulnerability explained more than 45% of the variance of dental anxiety, while negative dental experiences explained less than 1%. Instead, matching the two research traditions which attribute a central role in the development of dental anxiety to the real traumatic events or to the cognitive factors, Scandurra et al. [14] demonstrated that cognitive factors (specifically, the perception of having no control) mediate the relationship between traumatic dental events and dental anxiety, and that traumatic dental events continue to be a predictor of dental anxiety even in the absence of cognitive factors.

The only self-report tool existing in the literature and able to comprehensively measure cognitive factors in the development of dental anxiety according to the CVM is the *Dental Fear Maintenance Questionnaire* (DFMQ) [15]. The DFMQ is a scale of 32 items with Likert scale responses (from 1 “Strongly disagree” to 5 “Strongly in agreement”) that evaluates four cognitive vulnerability patterns associated with dental anxiety: dangerousness, differentiated into general dangerousness (e.g., painfulness of dental visits in general) and specific dangerousness (e.g., specific dangerous dental situations that could cause pain or harm); disgust (e.g., dislike of the smell and taste of blood and/or medications, or a dentist being unpleasant and not using gloves or a mask); unpredictability (e.g., fear of unpredictable events such as unexpected pain, complications, and treatments); and uncontrollability (e.g., fear of not being in control in the dental chair or feeling powerless when not involved in the decision-making process). Despite the central role that cognitive factors play in the development of dental anxiety, there are no self-report instruments on this topic in Italy, leaving Italian researchers and dentists in need of a measure to assess these dimensions. Therefore, the first objective of the study was to translate and validate the DFMQ scale in Italian.

In the present study, we assessed the psychometric characteristics of the Italian version of the DFMQ in a sample of dental patients by evaluating the model fit and different types of validity (i.e., predictive, convergent, construct, and discriminant validity). Specifically, we hypothesized that: (1) the five subscales of the DFMQ would have good fit indices in the Italian sample (i.e., model fit; Hypothesis 1); (2) each of the DFMQ subscales would correlate positively with dental anxiety (i.e., predictive validity; Hypothesis 2); (3) each of the DFMQ subscales would correlate positively with dental traumatic events (i.e., convergent validity; Hypothesis 3); (4) each of the DFMQ subscales would correlate positively with trait anxiety (i.e., construct validity; Hypothesis 4); and (5) in accordance with Kazdin’s recommendations for conceptual distinction among constructs [16], the correlations of the DFMQ with dental anxiety, dental traumatic events, and trait anxiety would be below 0.60 (i.e., discriminant validity; Hypothesis 5). Finally, we also examined, without specific hypotheses, which DFMQ dimensions were more strongly associated with high dental anxiety, and whether differences based on gender (men vs. women) and age ranges between DFMQ dimensions existed.

## 2. Materials and Methods

### 2.1. Procedures and Participants

In this study, we conducted a cross-sectional survey among dental patients who visited the Department of Dental Surgery at the University of Naples Federico II. The data analyzed in the current study were collected between June 2021 and June 2022. Participants were eligible to participate in the study if they: (1) were ≥ 18 years of age, the Italian age of consent; (2) spoke Italian; (3) were able to understand and sign the informed consent form; and (4) were able to complete the questionnaire independently. Written informed consent was obtained from all participants.

A total of 200 patients (90 men and 110 women) participated in the survey. The age of the participants ranged from 18 to 84 years (*M* = 51.92, *SD* = 19.11), and most of them had an educational level ≤ high school (*n* = 173; 86.5%).

The study was approved by the Ethical Medical Committee of the University of Naples Federico II (project identification code: 258/21; date of approval: 21 June 2021), conducted in accordance with the EU General Data Protection Regulation, and designed in respect of the principles of the Declaration of Helsinki on Ethical Principles for Medical Research Involving Human Subjects. The study was also conducted in accordance with the Strengthening the Reporting of Observational Studies in Epidemiology (STROBE) statement for cross-sectional studies.

### 2.2. Measures

#### 2.2.1. Cognitive Vulnerability to Dental Anxiety

Cognitive vulnerability to dental anxiety was assessed using the DFMQ [15]. The DFMQ is a scale of 32 items with Likert scale responses (from 1 “Strongly disagree” to 5 “Strongly in agreement”) that evaluates 4 cognitive vulnerability patterns associated with dental anxiety according to the CVM [1,13]. These are dangerousness, differentiated into general dangerousness (e.g., painfulness of dental visits in general) and specific dangerousness (e.g., specific dangerous dental situations that could cause pain or harm); disgust (e.g., dislike of the smell and taste of blood and/or medications, or a dentist being unpleasant and not using gloves or a mask); unpredictability (e.g., fear of unpredictable events such as unexpected pain, complications, and treatments); and uncontrollability (e.g., fear of not being in control in the dental chair or feeling powerless when not involved in the decision-making process). Responses were scored on a scale of 1 (strongly disagree) to 5 (strongly agree), with higher scores on the subscales indicating greater cognitive vulnerability.

#### 2.2.2. Dental Anxiety

Dental anxiety was measured using the Modified Dental Anxiety Scale (MDAS) [17], a 5-item scale designed to measure the severity of dental anxiety. Response options range from 1 to 5, with a total score ranging from 5 (not anxious) to 25 (extremely anxious). The cut-off score of 19 indicates a high level of dental anxiety. The Cronbach’s alpha coefficient for the current sample was 0.94.

#### 2.2.3. Traumatic Dental Experiences

The degree of distress related to traumatic dental experiences was assessed using the 16-item “Traumatic Dental Experiences” (TDE) subscale of the Level of Exposure-Dental Experiences Questionnaire (LOE-DEQ) [9,10,11,12,13,14,15,16,17,18]. On LOE-DEQ, participants must indicate whether they experienced a specific stressful event (yes or no). The Cronbach’s alpha coefficient for the current sample was 0.80.

#### 2.2.4. Trait Anxiety

Trait anxiety was assessed with the PROMIS Emotional Distress–Anxiety Short Form (PEDA-SF) [19], a 7-item version of the PROMIS Anxiety Short Form that evaluates the dominance of anxiety in subjects over 18 years of age. Responses are rated on a scale of 1 (never) to 5 (always), with higher scores indicating greater anxiety. The Cronbach’s alpha coefficient for the current sample was 0.95.

#### 2.2.5. DFMQ Translation

The translation of the DFMQ into Italian followed the back-translation method [20], in which five phases were carried out, namely: (1) each item of the DFMQ was independently translated from English into Italian by three experts in the field of dentistry and psychology; (2) the 3 Italian versions of the DFMQ were compared and agreement was reached on a final version (the first, second, and sixth authors); (3) the new Italian version of the DFMQ was translated into English by a native English speaker with excellent knowledge of Italian; (4) the new English version of the DFMQ was compared with the original by the fourth and seventh authors of the present paper, and agreement was reached on the final version; and (5) three independent researchers participated in a short online survey to evaluate the content and comprehensibility of each item. The instructions were, “How clear is the content of the following items?” on a five-point Likert scale (from “not at all clear” to “completely clear”); the average for all items was 4.52. The Italian version of the DFMQ can be found in Appendix A.

#### 2.2.6. Statistical Analyses

To assess model fit of the DFMQ, we performed a Confirmatory Factor Analysis (CFA) with Jamovi (version 2.3.0) (Sydney, Australia) using robust weighted least-square estimation (WLSM). Model fit was assessed by calculating estimated loadings and by the following indices: chi Square/degrees of freedom (χ^2^/df), root-mean-square error of approximation (RMSEA), standardized root-mean-square residual (SRMR), comparative fit index (CFI), incremental fit index (IFI), and Tucker–Lewis index (TLI). Based on the recommendations of Cole [21] and Kline [22], values of χ^2^/df < 2, RMSEA and SRMR < 0.08, TLI and CFI > 0.95, and IFI > 0.90 were considered to indicate a good fit to the data. The internal consistency reliability of each DFMQ subscale was calculated using the Cronbach’s alpha. Based on the suggestions of Muthén and Muthén [23], an appropriate sample size for a CFA model is approximately 150 participants.

In addition, we assessed the predictive, convergent, construct, and discriminant validity of the DFMQ by performing a series of correlations between the subscales of the DFMQ, dental anxiety, dental traumatic events, and trait anxiety using the Pearson correlation coefficient.

Furthermore, we assessed the associations between the DFMQ subscales and high dental anxiety by binary logistic regressions, considering the 5 subscale scores as explanatory variables, and high dental anxiety as a dichotomous dependent variable (≥19 vs. <19). We performed two types of analyses, as follows: (1) a univariate analysis, in which the DFMQ subscales were included individually; (2) a multivariate analysis, in which the DFMQ subscales were all included together to assess the estimated effect and the effect of each variable, holding all other variables constant. In both cases, the odds ratio (OR) was calculated with 95% confidence intervals (95%CI), accounting for the potentially confounding role of gender and age.

Finally, we assessed potential differences between DFMQ dimensions based on gender (men vs. women) and age ranges (18–30, 31–45, 46–60, 61+) by using Student’s *t*-test and one-way analysis of variance (ANOVA), respectively.

## 3. Results

### 3.1. Confirmatory Factor Analysis (CFA)

The original five-factor model of Topcu and Buchanan [15] was fit to the data obtained from the Italian sample, which confirmed our first hypothesis. Indeed, the following indices were found: χ^2^/df = 1.46, CFI = 0.962, RMSEA = 0.048 (CI = 0.039, 0.057), SRMR = 0.052, TLI = 0.953, and IFI = 0.968. The internal-consistency reliability calculated using the Cronbach’s alpha was adequate for each subscale, ranging from 0.71 to 0.92. Complete model statistics (i.e., standardized factor loadings, standard errors, Cronbach’s alpha, range, mean, and standard deviations) are presented in Table 1.

### 3.2. Predictive, Convergent, Construct, and Discriminant Validity

Correlational analyses for Hypotheses 2 to 5 are shown in Table 2. Regarding the predictive validity of the DFMQ (i.e., Hypothesis 2), all DFMQ subscales correlated positively with dental anxiety, indicating that higher scores on each indicator of cognitive vulnerability were associated with higher levels of dental anxiety. As regards the convergent validity of the DFMQ (i.e., Hypothesis 3), all DFMQ subscales correlated positively with dental traumatic events, suggesting that a higher cognitive vulnerability was associated with higher levels of dental traumatic events. As for the construct validity of the DFMQ (i.e., Hypothesis 4), all DFMQ subscales correlated positively with trait anxiety, suggesting that higher scores on each indicator of cognitive vulnerability were associated with a greater trait anxiety. Finally, regarding the discriminant validity of the DFMQ (i.e., Hypothesis 5), all correlations related to Hypotheses 2 through 4 were below 0.60, confirming this type of validity. Thus, all types of validity of the DFMQ were confirmed.

### 3.3. Associations of DFMQ Subscales with Dental Anxiety

A high percentage (n = 47; 23.5%) of participants were above the MDAS threshold (≥19), indicating the presence of severe dental anxiety. Neither gender nor age were found to be significant variables influencing dental anxiety (*p*s > 0.05). Therefore, these variables were not included as covariates in the logistic regression models.

Regarding the associations between the DFMQ subscales and high dental anxiety (Table 3), the results showed that all indicators of cognitive vulnerability were significantly associated with a high dental anxiety when each variable was included independently. In contrast, when the DFMQ subscales were considered together, only unpredictability and uncontrollability were found to be associated with a high dental anxiety. Specifically, an increasing unpredictability and uncontrollability was associated with an almost two-fold increase in the likelihood of dental anxiety.

### 3.4. Differences Based on Gender and Age between DFMQ Dimensions

The Student’s *t*-test performed to compare the potential differences between DFMQ dimensions based on gender showed that women presented higher levels of unpredictability, uncontrollability, and general dangerousness than men. On the other hand, no differences were found with respect to disgust and specific dangerousness. (Table 4) 

The one-way ANOVA performed to compare the effect of age on DFMQ dimensions revealed there were significant effects of age on unpredictability, uncontrollability, and specific dangerousness (Table 5). Post-hoc comparisons performed through Bonferroni corrections revealed that participants aged 18–30 years old had lower levels of unpredictability and uncontrollability than those aged 46–60 and 61+ years old (*p*s < 0.05), and lower levels of specific dangerousness than those aged 61+ and even those aged 46–60 years old. Thus, this group of results showed that older patients presented higher levels of some vulnerability cognitive patterns than younger patients.

## 4. Discussion

The aim of this study was to assess the psychometric characteristics of the Italian version of the DFMQ in a sample of dental patients by evaluating the model fit and different types of validity (i.e., predictive, convergent, construct, and discriminant validity). We also examined which DFMQ dimensions were more strongly associated with high dental anxiety, and whether differences based on gender (men vs. women) and age ranges between DFMQ dimensions existed. The results obtained through CFA demonstrated an appropriate fit to the data, confirming the original five-factor structure of the scale. To the best of our knowledge, this is the first questionnaire assessing cognitive vulnerability patterns in dental anxiety available for use in the Italian context. Thus, this study provides Italian researchers and dentists with a scale able to assess these dimensions in dental patients.

Regarding the predictive validity of the DFMQ, all DFMQ subscales correlated positively with dental anxiety, indicating that higher scores on each indicator of cognitive vulnerability were associated with higher levels of dental anxiety. However, when the DFMQ subscales were considered together, only unpredictability and uncontrollability were found to be associated with a high dental anxiety. These findings are consistent with previous studies that show that lack of control, which can be considered the latent factor of both unpredictability and uncontrollability, was positively associated with dental anxiety [24], representing the most significant predictor of dental anxiety [24,25]. In this context, the fear of losing control in the dental chair or feeling powerless if not involved in the decision-making process has been widely associated with the development of dental anxiety and represent a mediator between a negative dental experience and anxiety [14,26]. Indeed, it has been proven that different and mixed emotional experiences are a psychological vulnerability factor related to triggering and growing anxiety disorders, as well as the perception of not being able to control the situation [27]. In addition, panic disorders are among the various anxiety disorders recognized as resulting from an impaired perceived emotional control [28]. Other anxiety disorders include post-traumatic stress disorder [29] and generalized anxiety disorder [30]. A relationship between anxiety and environmental stressors could be moderated over time by the presence of such a vulnerability factor. Regarding the considerations of this study, the correlation between dental anxiety and the lack of a sense of control has been shown to be a key cognitive dimension in the development of dental anxiety [31].

Concerning the convergent validity of the DFMQ, all DFMQ subscales correlated positively with dental traumatic events, suggesting that a higher cognitive vulnerability was associated with higher levels of dental traumatic events. This finding is in line with the research tradition highlighting the significant role of past traumatic events within dental settings [14,25]. Indeed, although the most recent research tends to consider cognitive vulnerability patterns as the most predictive factors of dental anxiety [12,19,29], other studies have found that traumatic dental events may still be considered as significant predictors of the development of dental anxiety while, at the same time, attributing a central role to cognitive vulnerability [14].

As for the construct validity of the DFMQ, all DFMQ subscales correlated positively with trait anxiety, suggesting that higher scores on each indicator of cognitive vulnerability were associated with a greater trait anxiety. This finding is not surprising, as dental anxiety is a specific form of anxiety and, as such, people having a constitutional vulnerability to anxiety disorders are more likely to also report dental anxiety [28,30].

Finally, the comparison of DFMQ dimensions by gender showed that women exhibited higher levels of unpredictability, uncontrollability, and general dangerousness than men. In contrast, no differences were found in disgust and specific dangerousness. These findings can be interpreted in light of previous studies that found that women tend to have higher levels of dental anxiety than men [14,30,31,32,33,34,35,36]. Specifically, Carrillo-Diaz et al. [37] explained this finding by hypothesizing that women tend to overestimate the likelihood of danger and that this would elicit a stronger anxiety response. However, it is difficult to explain why no differences were found in disgust and specific dangerousness. Future studies might consider examining gender differences in cognitive vulnerability patterns through a qualitative approach.

Regarding age difference, there were significant effects of age on unpredictability, uncontrollability, and specific dangerousness. Specifically, younger participants had lower levels of the aforementioned vulnerability schemas than older counterparts, suggesting that older dental patients are at a higher risk of developing dental anxiety than younger patients. The results from previous studies on the association between age and dental anxiety are conflicting, with some reporting that younger people are more anxious than older people [38,39,40] and others reporting the opposite [41,42,43]. In our study, conducted with a clinical sample that had access to a public dental clinic, older patients showed higher levels of cognitive vulnerability patterns than their younger counterparts. These results can be correlated to the fact that public establishments receive older patients for full treatment protocols more frequently. In addition, elderly patients usually suffer from a systemic illness that places the patient in an anxious position out of fear for his or her life, which, in turn, affects dental treatments as collateral damage of their anxiety. Given these interpretive hypotheses and the fact that the results in the literature are contradictory, it would be interesting to qualitatively examine the relationship between age, cognitive vulnerability patterns, and dental anxiety.

The findings of the current study should be read considering some important limitations. First, its cross-sectional nature does not allow to draw any conclusive inferences about the temporality and causality of the relationships among the variables explored, specifically those related to the predictive validity. Thus, future studies should use a longitudinal research design to evaluate the cause–effect relationships between subscales of cognitive factors and dental anxiety. Second, the sample is not representative and was recruited at a single hospital, thus precluding the generalizability to the whole Italian context. Future Italian research should replicate this study analyzing potential socio-cultural differences by recruiting dental patients from diverse contexts, and should recruit larger and more diversified samples. Third, the use of questionnaires in clinical practice may not be entirely reliable. Questionnaires provide a relatively cheap, quick, and efficient way to obtain large amounts of information from a large sample of individuals. However, respondents may not be 100% truthful in their answers due to social desirability. Furthermore, there is a possibility that some questions may be ignored, left unanswered, or difficult to understand. Although the DFMQ questionnaire has been intended for adults, future research could focus on the adaptation and validation of DFMQ for dental pediatric patients as well.

## 5. Conclusions

Despite its limitations, this study allows Italian researchers and dentists to benefit from the use of the DFMQ in their practice. Indeed, our study demonstrated the importance of individuals’ cognitions and perceptions related to dental anxiety. Dentists should emphasize the sense of control of the patient, as well as the predictability and safety regarding the treatment, to prevent the development of dental anxiety. Future research could focus on clinical trials investigating whether the use of nonpharmacological methods of anxiety management, such as providing control and cognitive restructuring, can help to reduce dental anxiety and help clinicians to care for anxious patients when unpredictability and uncontrollability are high.

## Figures and Tables

**Table 1:** Confirmatory factor analysis of DFMQ.

Scale	Alpha	Range	Total Score: M (SD)
Disgust	0.90	1–5	2.72 (0.95)
Item			Std. Factor Loading (SE)
DFMQ 25			0.80 (0.97)
DFMQ 27			0.77 (0.07)
DFMQ 28			0.67 (0.07)
DFMQ 29			0.70 (0.07)
DFMQ 30			0.78 (0.08)
DFMQ 31			0.74 (0.08)
DFMQ 32			0.74 (0.08)
Unpredictability	0.92	1–5	2.96 (1.09)
Item			Std. Factor Loading (SE)
DFMQ 9			0.82 (0.07)
DFMQ 10			0.75 (0.07)
DFMQ 11			0.83 (0.08)
DFMQ 12			0.70 (0.08)
DFMQ 13			0.73 (0.07)
DFMQ 14			0.61 (0.09)
DFMQ 16			0.80 (0.07)
Uncontrollability	0.91	1–5	2.52 (0.99)
Item			Std. Factor Loading (SE)
DFMQ 2			0.74 (0.08)
DFMQ 3			0.76 (0.08)
DFMQ 4			0.74 (0.08)
DFMQ 5			0.74 (0.07)
DFMQ 6			0.82 (0.07)
DFMQ 7			0.84 (0.06)
DFMQ 8			0.77 (0.08)
Specific dangerousness	0.89	1–5	2.46 (0.93)
Item			Std. Factor Loading (SE)
DFMQ 19			0.59 (0.07)
DFMQ 20			0.73 (0.07)
DFMQ 21			0.81 (0.07)
DFMQ 22			0.66 (0.08)
DFMQ 23			0.84 (0.07)
DFMQ 24			0.82 (0.06)
DFMQ 26			0.74 (0.07)
General dangerousness	0.71	1–5	2.79 (1.02)
Item			Std. Factor Loading (SE)
DFMQ 17			0.77 (0.08)
DFMQ 18			0.58 (0.08)

*Notes*: DFMQ—Dental Fear Maintenance Questionnaire; M—Mean; SD—Standard Deviation; Std.—Standardized; SE—Standard Error.

**Table 2:** Correlations between DFMQ subscales, MDAS, TDE, and PEDA-SF.

Scales	1	2	3	4	5	6	7	8
1. Disgust	1.00							
2. Unpredictability	0.62 ***	1.00						
3. Uncontrollability	0.67 ***	0.67 ***	1.00					
4. Specific dangerousness	0.76 ***	0.65 ***	0.75 ***	1.00				
5. General dangerousness	0.60 ***	0.67 ***	0.63 ***	0.67 ***	1.00			
6. MDAS	0.45 ***	0.50 ***	0.52 ***	0.44 ***	0.41 ***	1.00		
7. TDE	0.15 *	0.24 **	0.12 *	0.14 *	0.17 *	0.32 ***	1.00	
8. PEDA-SF	0.43 ***	0.45 ***	0.49 ***	0.47 ***	0.41 ***	0.74 ***	0.30 ***	1.00

*Notes*: DFMQ—Dental Fear Maintenance Questionnaire; MDAS—Modified Dental Anxiety Scale; TDE—Traumatic Dental Experiences; PEDA-FS—PROMIS Emotional Distress–Anxiety Short Form. * *p* < 0.05; ** *p* < 0.01; *** *p* < 0.001.

**Table 3:** Logistic regression of DFMQ on high dental anxiety.

	Univariate Analysis		Multivariate Analysis	
	OR (95%CI)	*p*	OR (95%CI)	*p*
Disgust	2.87 (1.85, 4.45)	<0.001	1.79 (0.93, 3.47)	0.083
Unpredictability	3.23 (2.03, 5.13)	<0.001	1.92 (1.08, 3.42)	0.026
Uncontrollability	2.98 (1.96, 4.53)	<0.001	1.90 (1.03, 3.51)	0.039
Specific dangerousness	2.26 (1.53, 3.35)	<0.001	0.62 (0.31, 1.24)	0.174
General dangerousness	2.33 (1.58, 3.41)	<0.001	1.09 (0.63, 1.88)	0.753

*Notes*: DFMQ—Dental Fear Maintenance Questionnaire; OR—Odds Ratio; CI—Confidence Intervals; *p*—*p*-value.

**Table 4:** Comparison of means between men and women with respect to DFMQ dimensions.

	Men (*n* = 90)	Women (*n* = 110)				
	*M* (*SD*)	*M* (*SD*)	*t*	*p*	95% *CI*	*d*
Disgust	2.63 (0.93)	2.79 (0.96)	−1.20	0.233	−0.43, 0.10	0.17
Unpredictability	2.74 (1.02)	3.14 (1.12)	−2.61	0.010	−0.70, −0.09	0.37
Uncontrollability	2.37 (0.94)	2.64 (1.03)	−1.95	0.048	−0.55, 0.01	0.27
Specific dangerousness	2.38 (0.90)	2.51 (0.95)	−0.99	0.324	−0.39, 0.13	0.14
General dangerousness	2.64 (1.01)	2.92 (1.03)	−1.99	0.047	−0.57, −0.01	0.27

*Notes*: DFMQ—Dental Fear Maintenance Questionnaire; M—Mean; SD—Standard deviation; t—Student’s *t*-test; *p*—*p*-value; CI—Confidence interval; d—Cohen’s d.

**Table 5:** Comparison of means between different age ranges with respect to DFMQ dimensions.

	*N*	*M* (*SD*)	95% *CI*	*F*	*p*
Disgust					
18–30 years	43	2.42 (0.89)	2.15, 2.71	2.56	0.057
31–45 years	24	2.60 (0.84)	2.25, 2.95		
46–60 years	51	2.93 (0.94)	2.67, 3.20		
61+ years	82	2.78 (0.98)	2.56, 2.99		
Unpredictability					
18–30 years	43	2.53 (0.86)	2.26, 2.79	3.12	0.027
31–45 years	24	2.94 (1.02)	2.51, 3.38		
46–60 years	51	3.13 (1.11)	2.82, 3.44		
61+ years	82	3.09 (1.17)	2.83, 3.35		
Uncontrollability					
18–30 years	43	2.04 (0.83)	1.78, 2.29	4.47	0.005
31–45 years	24	2.56 (0.76)	2.24, 2.89		
46–60 years	51	2.68 (1.15)	2.36, 3.01		
61+ years	82	2.65 (0.98)	2.43, 2.87		
Specific dangerousness					
18–30 years	43	2.11 (0.88)	1.84, 2.38	2.65	0.049
31–45 years	24	2.52 (0.77)	2.19, 2.84		
46–60 years	51	2.49 (0.96)	2.22, 2.76		
61+ years	82	2.59 (0.94)	2.39, 2.80		
General dangerousness					
18–30 years	43	2.56 (0.95)	2.26, 2.85	1.23	0.299
31–45 years	24	2.89 (1.03)	2.46, 3.33		
46–60 years	51	2.76 (1.15)	2.44, 3.09		
61+ years	82	2.92 (0.97)	2,70, 3.13		

*Notes*: M—Mean; SD—Standard deviation; CI—Confidence Interval; *p*—*p*-value.

## Data Availability

Data is unavailable due to privacy or ethical restrictions.

## Appendix A

**Italian Version of the DFMQ**

*Per favore, indichi il grado con cui si trova in accordo o in disaccordo con le seguenti affermazioni. Nel rispondere, utilizzi la seguente scala:*

**1**	**2**	**3**	**4**	**5**				
Fortemente in disaccordo	In disaccordo	Né d’accordo né in disaccordo	D’accordo	Fortemente d’accordo				

1. Non sento di avere il controllo quando sono sulla poltrona del dentista				1	2	3	4	5
2. Non sento di poter dire al dentista di fermarsi se sono agitato/a				1	2	3	4	5
3. Mi sento impotente quando sono sulla poltrona del dentista				1	2	3	4	5
4. Sento che il dentista farà ciò che vuole senza preoccuparsi di ciò che dico io				1	2	3	4	5
5. Non sento di potermi fermare per riposare durante il trattamento se ne sento la necessità				1	2	3	4	5
6. Mi sento come intrappolato/a sulla poltrona del dentista				1	2	3	4	5
7. Non sento di poter essere coinvolto/a nel decidere quale trattamento vorrei mi fosse fatto				1	2	3	4	5
8. Mi sento vulnerabile quando sono sdraiato/a sulla poltrona del dentista				1	2	3	4	5
9. Quando sono sulla poltrona del dentista sento di non sapere cosa succederà dopo				1	2	3	4	5
10. Sento di non conoscere l’intensità del dolore che potrei provare durante il trattamento				1	2	3	4	5
11. Sento di non sapere cosa il dentista farà (ad es., iniezione, uso del trapano) mentre mi sottopongo a procedure odontoiatriche (ad es., devitalizzazione)				1	2	3	4	5
12. Sento di non sapere quale strumento odontoiatrico sceglierà il dentista e a cosa servirà				1	2	3	4	5
13. Sento di non sapere se avrò dolore dopo il trattamento				1	2	3	4	5
14. Sento di non sapere se avrò qualche complicanza dopo il trattamento				1	2	3	4	5
15. Sento di non sapere se perderò qualche dente durante il trattamento				1	2	3	4	5
16. Sento di non sapere cosa sta succedendo nella mia bocca				1	2	3	4	5
17. Credo che potrei essere ferito/a quando sono sulla poltrona del dentista				1	2	3	4	5
18. Credo di poter sentire dolore quando vado dal dentista				1	2	3	4	5
19. Credo di poter avere delle complicanze (infezioni, sanguinamento eccessivo) dopo e durante le procedure odontoiatriche				1	2	3	4	5
20. Credo che al dentista possa cadere dalle mani il trapano.				1	2	3	4	5
21. Credo che il dentista possa effettuare dei trattamenti non necessari				1	2	3	4	5
22. Ho paura che l’anestesia non faccia effetto.				1	2	3	4	5
23. Credo di poter soffocare durante il trattamento odontoiatrico				1	2	3	4	5
24. Credo che gli strumenti usati possano rimanere bloccati in bocca				1	2	3	4	5
25. Trovo sgradevole l’odore dello studio dentistico				1	2	3	4	5
26. Credo di poter essere a rischio di contrarre infezioni.				1	2	3	4	5
27. Trovo sgradevole il sapore e/o l’odore delle medicazioni usate durante le procedure odontoiatriche				1	2	3	4	5
28. Trovo sgradevole sputare sangue e/o saliva				1	2	3	4	5
29. Trovo sgradevole l’odore e/o il sapore del sangue				1	2	3	4	5
30. Trovo sgradevole avere le mani del dentista in bocca.				1	2	3	4	5
31. Trovo sgradevole l’odore e/o il sapore dello smalto del dente bruciato dal trapano.				1	2	3	4	5
32. Trovo sgradevole l’acqua nebulizzata in bocca				1	2	3	4	5
